# Methylene blue as an early diagnostic marker for oral precancer and cancer

**DOI:** 10.1186/2193-1801-2-95

**Published:** 2013-03-09

**Authors:** Akhtar Riaz, Balasundari Shreedhar, Mala Kamboj, S Natarajan

**Affiliations:** Department of Oral Pathology and Microbiology, Career Post Graduate Institute of Dental Sciences and Hospital, C-1111, Indiranagar, Lucknow, 226016 India

**Keywords:** Methylene blue, Oral precancer, Oral cancer, Early marker

## Abstract

Oral cancer is one of the most common neoplasm’s and is ranked eighth in the cancer incidence worldwide. Early detection is of critical importance because survival rates markedly improve. *In vivo* staining is a simple, inexpensive, and fairly sensitive method. Involved 120 patients (50 with Premalignant Lesion, 50 with OSCC and 20 controls) stained by Methylene Blue (MB). The results of MB uptake were compared with a simultaneous biopsy of these lesions. Pathologically confirmed precancers and cancers were the positive targets of this screening, while hyperkeratosis without dysplasia and no evidence of malignancy were sorted as negative subjects of screening. The results revealed sensitivity of 91.4%, specificity of 66.6%, positive predictive value 97.7% and negative predictive value 33% leading to diagnostic accuracy of MB stain to 90%. We state that MB staining is useful diagnostic tool in community oral cancer screening programmes for high-risk individuals.

**Electronic supplementary material:**

The online version of this article (doi:10.1186/2193-1801-2-95) contains supplementary material, which is available to authorized users.

## Introduction

Oral cavity cancer accounts for approximately 3% of all malignancies and is a significant worldwide health problem (Kademani 2007; Silverman 2001). Most oral malignancies occur as squamous cell carcinomas; despite remarkable advances in treatment modalities, the 5-year survival rate has not significantly improved over the past several decades and still hovers at about 50–60% (Ries et al. 2999). Oral squamous cell carcinoma (OSCC) is one of the most common neoplasm and it is ranked eighth in the cancer incidence worldwide, with an annual incidence rate of 64,460. However total number of cases at any given time will be 2.5 to 3 times higher than this number (Mehta & Hamner 1993).

Many OSCCs develop from premalignant lesions & conditions of the oral cavity (Silverman et al. 1984; Silverman 1968). A wide array of conditions have been implicated in the development of oral cancer, including leukoplakia, erythroplakia, palatal lesion of reverse cigar smoking, oral submucous fibrosis, discoid lupus erythematosus, and hereditary disorders such as dyskeratosis congenita and epidermolysis bullosa (Warnakulasuriya et al. 2007). Despite the general accessibility of the oral cavity during physical examination, many malignancies are not diagnosed until late stages of disease. In order to prevent malignant transformation of these precursor lesions, multiple screening and detection techniques have been developed to address this problem. The early detection of cancer is of critical importance because survival rates markedly improve when the oral lesion is identified at an early stage (Ries et al. 2999). Periodic clinical examination of the oral cavity is the key to early detection of oral cancer (Ya-Wei 2007a). Among the diagnostic tool, *in vivo* staining is advocated as a simple, inexpensive and fairly sensitive method (Ya-Wei 2007b).

Methylene Blue (MB) has been used to detect gastric, prostrate and bladder cancers (Mufti et al. 1990; Gill et al. 1984; Yu et al. 1990). In the diagnosis, accuracy of the MB technique is used for identification of intestinal metaplasia, carcinoma or dysplasia. In one of the studies, its validity in detection of oral cancer and precancerous lesion has also been examined. The exact mechanism for the uptake of methylene blue in epithelial tissue may resemble that of toluidine blue in the acidophilic characteristic of cells with abnormal concentration of nucleic acid, resulting in differential uptake between normal/benign and highly dysplastic /malignant cells. The 90% sensitivity of MB was no less than 72–100% sensitive report with toluidine blue staining. Considering the low toxicity and cheaper prices, methylene blue may be conveniently substituted for large scale screening in high risk patients (Ya-Wei 2007a). Methylene blue is a commonly used stain that helps us see microscopic life in brilliant color. The dye, methylene blue shows the deepest shade of blue, indicating a strong attraction to acids including DNA.

In the present paper an attempt has been made to establish MB as an early diagnostic marker in the suspected cases of oral precancerous lesions and oral squamous cell carcinoma for wider screening programmes.

## Methods

### Subjects

The study group consisted of 120 subjects of both the sexes, 50 subjects with clinically suspicious premalignant lesions, 50 subjects with clinically suspicious oral cancers and 20 cases of subjects with clinically appearing normal mucosa. A provisional diagnosis of leukoplakia, erythroplakia, smoker’s palate (premalignant lesions) and Oral cancer were made on basis of clinical examination. Patient’s more than or equal to 15 yrs of age, with the habit of smoking and/or tobacco consumption were made part of the study. Patients with age less than 15 years, immunocompromised states, with premalignant conditions, and patients with diabetes or other systemic disorders were excluded from the study. Ethics committee approval from the college ethics committee was taken for the smooth conduct of the study. (CPGIDSH/09). Controls were subjects with clinically normal appearing oral mucosa. The subjects were made to sit comfortably on the dental chair and were thoroughly examined under artificial illumination. The clinical examination was done and the relevant data was entered into the proforma.

### Gargling solution

Methylene blue dye system had 2 solution bottles. The dye rinse solution (Bottle A) had 1% methylene blue, 1% malachite, 0.5% eosin, glycerol, and dimethylsulfoxide. Pre- and post-rinse solution (Bottle B) had 1% lactic acid, and purified water.

### Staining procedure

The application of methylene blue was as follows. The patients were directed to rinse their mouth with 1% lactic acid & distilled water for 30 seconds to remove food debris and excess saliva and to provide a consistent oral environment. The mucosa of the target area was gently dried with gauze and power air spray to ensure that the lesion was not being contaminated with saliva. The dye was directly applied on the lesion with help of cotton bud (Mufti et al. 1990) first and after used as a mouth rinse gargle (Ya-Wei 2007a) with Methylene blue for 30 seconds; then expectorated. Patients then rinsed again with 1% lactic acid for 30 seconds to wash out the excess dye (Ya-Wei 2007a). The pattern of dye retention was assessed by the intensity of stain retention on the lesion. Local, and deep blue stains were marked as positive (+) reaction. Wide, shallow or faint blue stains were marked as negative (–) reaction (Ya-Wei 2007a). The results of methylene blue dye staining were recorded with photographs and incisional biopsy was performed simultaneously in the suspected lesions to compare the accuracy of the diagnostic capability of methylene blue.

### Biopsy

Incisional biopsy was performed in the most obvious staining area of the suspected lesion of the patient under local anaesthesia. The specimen was then fixed in 10% neutral buffered formalin and processed in the oral pathology laboratory for initial routine pathologic diagnosis.

### Histological examination

All the specimens were microscopically evaluated by pathologists who were blind to the results of methylene blue stain. The pathology reports of the lesions were classified as A) Oral Precancerous: Mild, Moderate and Severe Dysplasia B) Oral Cancer: Well, Moderately and Poorly differentiated Squamous Cell Carcinoma C) Lesions neither precancerous nor malignant: Hyperkeratosis without dysplasia and No evidence of malignancy. The results were then deduced and were statistically analyzed using the various statistical tests given below and the final results were drawn.

### Statistical analysis

The pathologically proven OSCC and precancerous lesions were the targets of screening and the concerned is presented by number and percentage of the same. The results of positive/negative uptake of methylene blue in each lesion were correlated with the histopathological diagnosis. Statistical analysis was performed, including sensitivity, specificity, positive and negative predictive values. The association of methylene blue uptake and pathologic diagnosis among the precancers/ OSCC group, lesions neither premalignant nor malignant group, nor normal group were analyzed using Fisher’s exact test (Wilson 1927). A p value (probability value) of less than 0.01 was considered very significant. The statistical analysis was done using Critical Appraisal Diagnostic Test and GraphPad Software.

## Results

### Subject characteristics

120 subjects (50 with Premalignant Lesion, 50 with OSCC and 20 controls) were enrolled in this study. The patient’s ages (patient group) ranged from 15 yrs to 80 yrs, with the ratio of male to female being 5:1. Both the groups gave history of tobacco consumption. The suspected lesions were distributed over the buccal mucosa (n = 84), palate (n = 6), tongue (n = 4), floor of the mouth (n = 2). In the control group, as methylene blue dye was not used to examine the oral cavity, it was necessary to verify that the dye would not be retained on normal mucosa. The results demonstrated that there was no retained dye in the control group.

### Methylene blue staining related to grade of pathology

**Figure 1:**
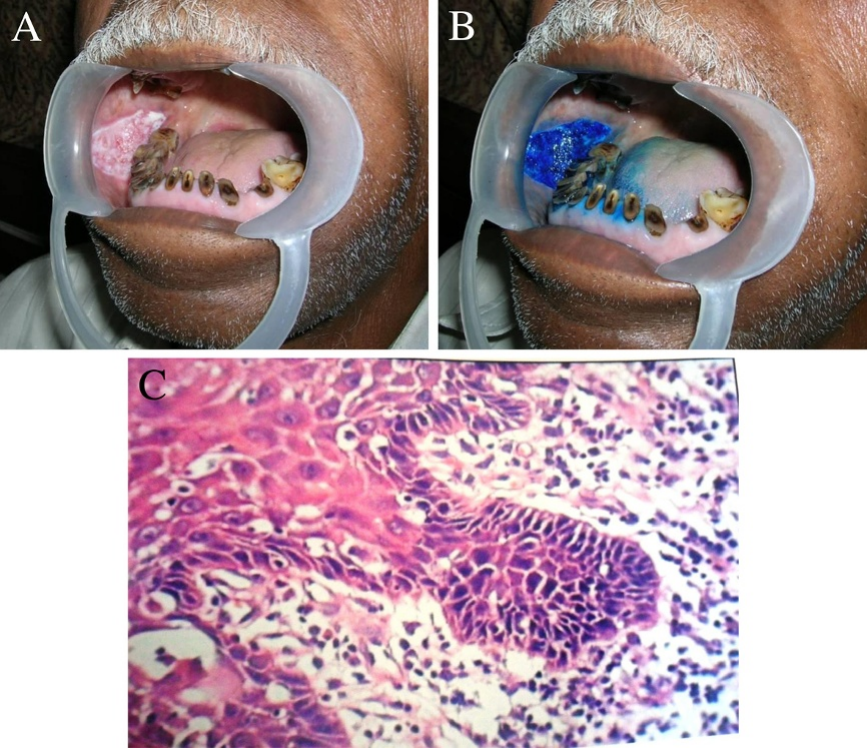
**Presentation of a true-positive staining on a red and white homogenous patch on the right buccal mucosa.** (**A**) The lesion presented clinically as a red and white homogenous patch. (**B**) Vital staining with methylene blue showed deep and focal staining of the lesion. (**C**) The final pathology revealed a severe dysplasia. (H &E, 40×).

**Figure 2:**
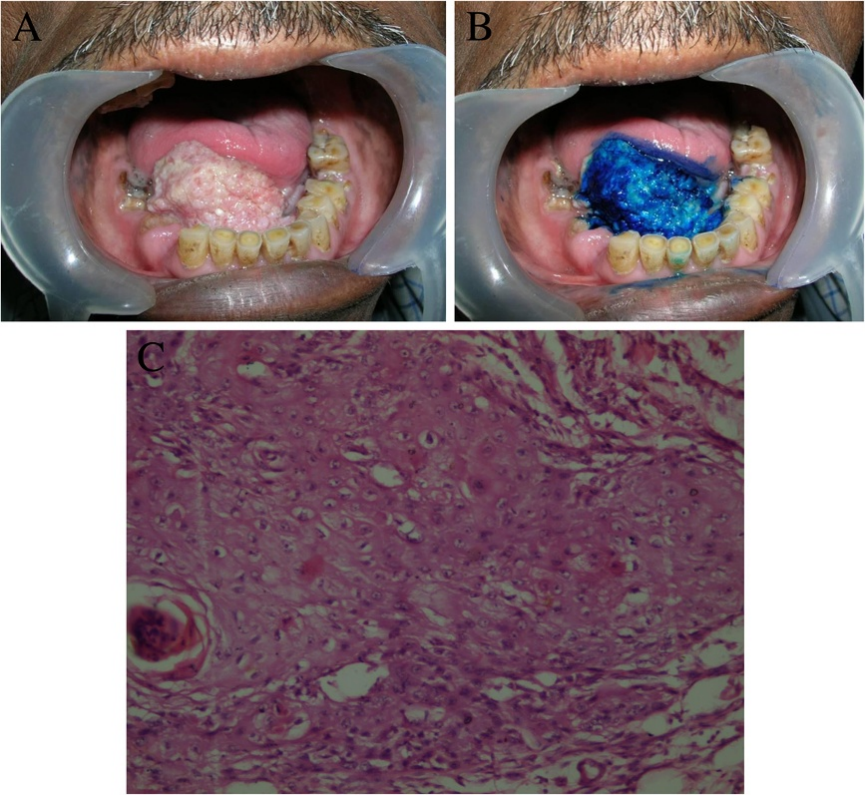
**Presentation of a true-positive staining on the floor of the mouth.** (**A**) The lesion presented clinically as a huge growth on the floor of the mouth with indurated margins. (**B**) Vital staining with methylene blue showed deep and focal staining of the lesion. (**C**) The final pathology revealed a moderately differentiated squamous cell carcinoma. (H &E, 40×).

The Clinical and Histological characteristics of the various study Groups are shown in Table 1. The pathologic grade was classified as precancer lesions, oral cancer lesions, hyperkeratosis without dysplasia and no evidence of malignancy. The following statistical terms were used to describe and analyze the relationship between the grade of pathology and the uptake of methylene blue staining. Sensitivity represents the proportion of histologically proved cancer/precancerous lesions which are detected by positive methylene blue staining. In the current study, 44 of 48 pathologically proven cancers and 42/46 precancerous lesions were positive with deep and focal methylene blue staining (Figure 1A,B,C, Figure 2A,B,C). The overall sensitivity was 91.4%.

**Table 1:** **Clinical and histological characteristics of the various study groups**

Criteria	Group I	Group II	Group III
	Premalignant lesions (n = 50)	OSCC (n = 50)	Controls (n = 20)
**Age ≤ 40 yrs**	28 56%	8 16%	12 60%
**≥40 yrs**	22 44%	42 84%	8 40%
**Sex Females**	2 4%	14 28%	4 20%
**Males**	48 96%	36 72%	16 80%
**Site Buccal Mucosa(BM)**	40 leukoplakia	44 BM with vest. ridge	----
**Palate**	4 smoker’s palate	2	
**Tongue**	2 leukoplakia	2	
**Floor of mouth**		2	
**Tobacco Use No**	0	0	20
**Yes**	50	50	0
**Histological Diagnosis**	Mild 10 20%	WDSCC 30 60%	---
	Moderate 34 68%	MDSCC 16 32%	
	Severe 2 4%	PDSCC 2 4 %	
	Hyperkeratosis 4 8%	No evidence of 2 4%	
	without dysplasia	Malignancy	

Specificity suggests the proportion of pathologic benign lesions, neither precancerous lesions nor cancers, which are correctly identified as negative staining of methylene blue. In our study, 4 out of 6 cases with no dysplasia or malignancy showed negative staining; thus, the specificity was 66.6%. The results of staining with methylene blue for all lesions correlated well with the pathologic diagnosis and are summarized in Table 2. Fisher’s exact test showed significant differences among cancer/precancerous lesions, lesions without dysplasia or malignancy, and normal control groups (p < 0.001). Overall, the positive predictive value was 97.7%, and the false predictive value was 33.3%.

**Table 2:** **Diagnostic accuracy of methylene blue in the study groups**

Methylene Blue Retention	Histological diagnosis				Overall (PML + SCC)
	Premalignant Lesion (PML)		Squamous Cell Carcinoma (SCC)		
	Dysplasia	Hyperkeratosis without dysplasia	OSCC	No evidence of malignancy	
**+**	42	1	44	1	
**−**	4	3	4	1	
**Sensitivity**	91.3%		91.6%		91.4%
**Specificity**	75%		50%		66.6%
**Positive predictive value**	97.6%		97.7%		97.7%
**Negative Predictive Value**	42.8%		20%		33.3%
**Diagnostic Accuracy**	90%		90%		90% **p < 0.0001(very significant)**

### Overall diagnostic validity of methylene blue stain

Out of 100 lesions, 88 (92%) lesions retained methylene blue stain while 12 (8%) lesions failed to retain the stain. Out of these 88 methylene blue stained lesions, 86 were histologically proved as having either dysplastic or carcinomatous change, while 2 (2%) were diagnosed as hyperkeratosis without dysplasia / no evidence of malignancy. Out of 12 unstained lesions 8 were histopathologically diagnosed as carcinomatous while 4 were diagnosed as hyperkeratosis without dysplasia / no evidence of malignancy respectively. The sensitivity of methylene blue in determining dysplastic and carcinomatous changes was determined as 91.4% while the specificity was determined as 66.6%, the positive predictive value 97.7%., the negative predictive value 33.3%. The overall diagnostic value of methylene blue stain in distinguishing premalignant and malignant lesion 90% The p value less than <0.001 which is indicates that these results are stastically very significant.

## Discussion

Oral Cancer is very common in a country like India due to habits of smoking and chewing tobacco containing pan masala. Tobacco consumption is the main etiologic factor inducing carcinogenesis in oral mucosa. WHO indicates a 500 patients increase in cancer by 2025 of which 220 will be due to tobacco use. A survey by WHOSEA (Women’s Health in South East Asia) has indicated that almost 50% of oral cancer in men and 25% cases of women in India are believed to be tobacco-borne (Mehta & Hamner 1993). Thus oral screening of high-risk individuals is very important in these countries. Amongst the various diagnostic tools, in vivo staining is advocated as simple and sensitive method for early detection of oral cancer (Ya-Wei 2007b).

For a large scale community screening, some dye materials easily help to identify abnormal mucosa tissue which raise oral examiners attention and refer the patients with suspicious lesions to oral surgeons for further examination. Toluidine Blue has been championed in many parts of world for several decades as a means of identifying clinically occult lesions in patients whose oral mucosa may otherwise be normal that is as a screening test or adjunct (Mashberg 1983). The efficacy of this technique has been evaluated in many reports with diverse results. It has yielded sensitivities between 72–100% and specificities between 45–67% in detecting suspicious malignancies (Epstein et al. 1997; Onofre et al. 2001; Epstein et al. 1992; Warnakulasuriya & Johnson 1996). However the material data safety sheet indicates that toluidine blue is probably toxic by ingestion and is seldom used in detecting cancers in other parts of body (Ya-Wei 2007a).

Methylene blue is another recently proposed dye for in vivo staining used in endoscopic examination (Bruno 2003). Its application has been reported recently in detecting some gastrointestinal abnormalities such as Barrett’s esophagus (Canto et al. 1996; Canto et al. 2000), gastric cancer (Canto et al. 2000), prostate cancer (Mufti et al. 1990; Gill et al. 1984) bladder Methylene blue (MB) is an acidophilic dye, it binds to the double helical DNA with a high affinity, as deduced from the absorption and fluorescence spectral data. Extensive hypochromism and red shifts in the absorption spectra were observed when MB binds to calf thymus DNA (CT DNA), which suggested the intercalation mechanism of MB into DNA bases. Upon binding to DNA, the fluorescence from MB was efficiently quenched by the DNA bases, with no shifts in the emission maximum. The large increases in the polarization upon binding to CT DNA supported the intercalation of MB into the helix (Wen-You et al. 2000). The binding of methylene blue to DNA and chromatin treated in various ways was also examined by equilibrium dialysis. The maximum r value (moles of bound dye/mole of nucleotide) was 1.0 for DNA, 0.6 for unfixed chromatin, and 0.83 for chromatin fixed in methanol-acetic acid104. All these studies have established the fact that methylene show proportionate binding to DNA nucleotide and this accounts for increase in intensity of stain color with increase in chromatin material in premalignant and malignant cells.

Very few studies have been conducted till now to establish the usage of methylene blue technique in detecting oral precancerous/cancerous lesions8. Screening studies should always be evaluated with respect to their sensitivity, specificity and predictive values (Lingen et al. 2008). Although there is no defined value for the ideal screening test but in general, it is desirable to have both high specificity (few false positives) and high sensitivity (few false negatives) (Lingen et al. 2008). In the present study, the pool of 100 patients (50 cancerous, 50 precancerous) were screened against 20 control (normal subjects) and we found that 88/94 pathologically proven precancers/cancerous lesions showed positive staining with localized and deep blue stain. Overall 91.4% sensitivity (91.3% for premalignant and 91.6% for OSCC) was reported with a false negative rate of 8.6%. Previous studies have indicated sensitivity of 72–100% (Epstein et al. 1997; Onofre et al. 2001; Epstein et al. 1992; Warnakulasuriya & Johnson 1996) in others and 90% in oral screening (Ya-Wei 2007a), on the basis of these and our studies we can say that using methylene blue dye for diagnostic screening is highly acceptable. For few false negatives results, we consider that ambiguous faint blue shallow stains, which may be misinterpreted as negatives but clinically suspicious of malignancy, are proved pathologically after biopsy. As far as specificity is concerned we obtained overall specificity of 66.6% (4/6) (75% in premalignant and 50% in OSCC) with a resulting 2 false positives, that were the cases of chronic unhealed ulcers. The high false positives rate was discussed to be related to the retention of stain in inflamed and trauma areas (Mashberg 1983). Other factors can be irregular, papillary or digital surfaces of the lesions, which may cause the mechanical retention of dye, contamination of saliva and plaque, retention of dye material in papilla of the tongue or minor salivary gland ducts over the mucosa (Ya-Wei 2007a).

Applying this method for screening high risk patients having habits of tobacco chewing or smoking, a large group of individuals may include those with obvious oral lesions and those with normal oral mucosa. To study these people and to re-evaluate the efficacy of lesions, a large population of the people with normal oral mucosa will lower the rate of false positives and result in higher specificity as found on our case where when compared with control (n = 20) false positives were 0 and specificity was 100% although our control group was having normal mucosa but the flaw in our experimental design was that these subjects were not having any type of tobacco chewing or smoking habits, as we cannot screen individuals with these habits and no lesions because performing biopsy in normal mucosa will be unethical. Thus we can say that this study has established methylene blue as a diagnostic agent (with diagnostic accuracy of 90% and stastically very significant results with p-value < 0.001) for early detection of cancerous and precancerous lesions. This dye will be convenient to substitute for toluidine blue because of its non-toxicity and low cost. However pathology report based on biopsy will always remain gold standard to exactly and accurately diagnose the lesion before a treatment modality is determined (Mashberg 1980).

Staining should be routinely used as a method to assist the choice of biopsy site and in the follow up of premalignant lesions and in the experienced hands marginal demarcation of the malignant lesions enables an intervention method to be adopted earlier for the diseases, which carries a high rate of morbidity and mortality. Further study is recommended to study the exact binding mechanism of methylene blue in precancerous, cancerous, benign and normal mucosa to establish this dye as the efficient diagnostic tool for diagnosing and differentiating between different pathological conditions.

## Authors’ original submitted files for images

Below are the links to the authors’ original submitted files for images. Authors’ original file for figure 1 Authors’ original file for figure 2
