# Supplementary figures and images for: Methylene blue as an early diagnostic marker for oral precancer and cancer
Source: Springerplus. 2013 Mar 9;2(1):95. doi: 10.1186/2193-1801-2-95 (PMC3602613; doi:10.1186/2193-1801-2-95)

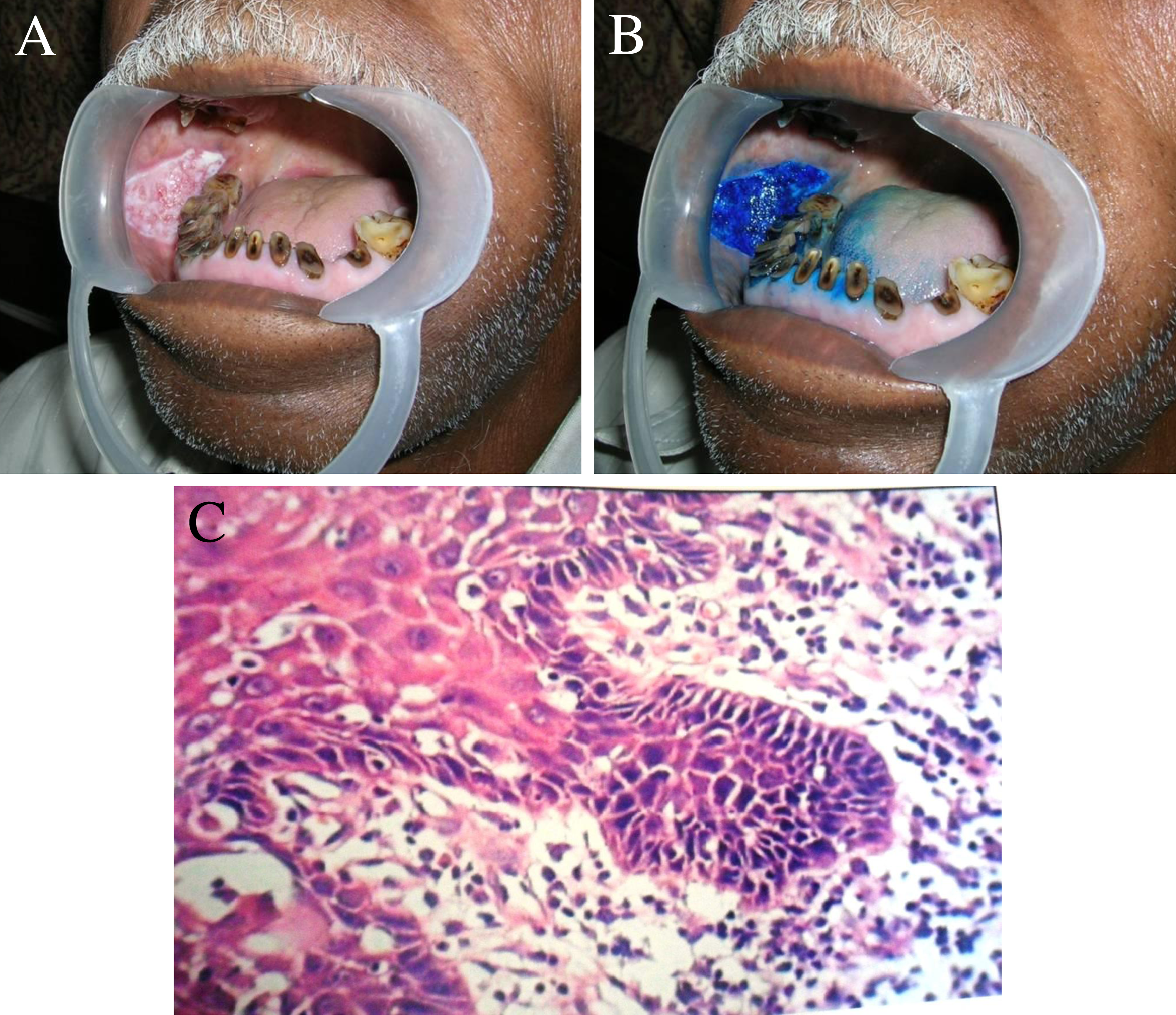

Supplement: Supplementary file 1 — Authors’ original file for figure 1 [file 40064_2013_153_MOESM1_ESM.tiff]

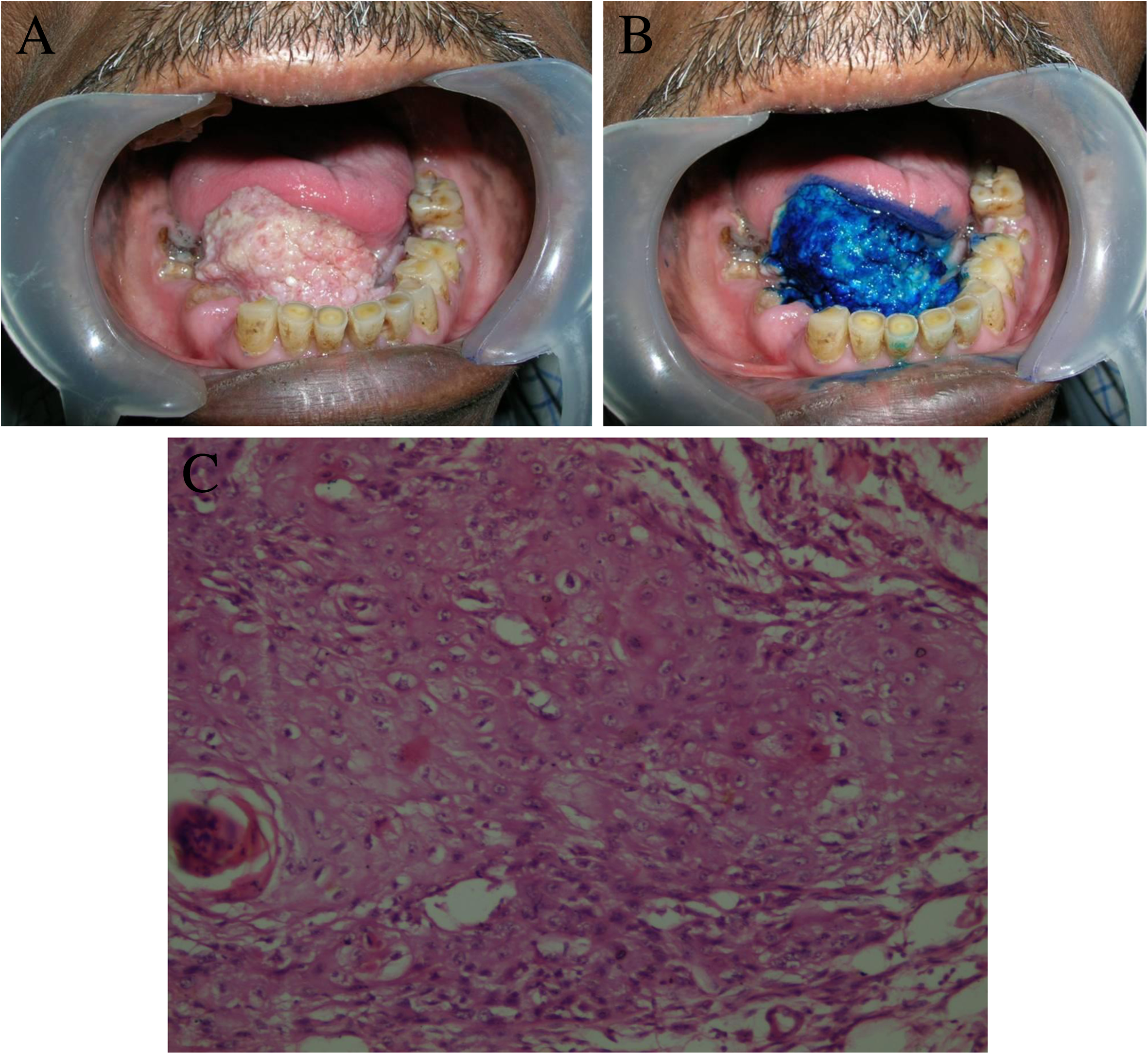

Supplement: Supplementary file 2 — Authors’ original file for figure 2 [file 40064_2013_153_MOESM2_ESM.tiff]
